# Hierarchical clustering by patient-reported pain distribution alone identifies distinct chronic pain subgroups differing by pain intensity, quality, and clinical outcomes

**DOI:** 10.1371/journal.pone.0254862

**Published:** 2021-08-04

**Authors:** Benedict J. Alter, Nathan P. Anderson, Andrea G. Gillman, Qing Yin, Jong-Hyeon Jeong, Ajay D. Wasan

**Affiliations:** 1 Department of Anesthesiology and Perioperative Medicine, University of Pittsburgh, Pittsburgh, Pennsylvania, United States of America; 2 Department of Biostatistics, University of Pittsburgh, Pittsburgh, Pennsylvania, United States of America; 3 Department of Psychiatry, University of Pittsburgh, Pittsburgh, Pennsylvania, United States of America; University of Würzburg, GERMANY

## Abstract

**Background:**

In clinical practice, the bodily distribution of chronic pain is often used in conjunction with other signs and symptoms to support a diagnosis or treatment plan. For example, the diagnosis of fibromyalgia involves tallying the areas of pain that a patient reports using a drawn body map. It remains unclear whether patterns of pain distribution independently inform aspects of the pain experience and influence patient outcomes. The objective of the current study was to evaluate the clinical relevance of patterns of pain distribution using an algorithmic approach agnostic to diagnosis or patient-reported facets of the pain experience.

**Methods and findings:**

A large cohort of patients (N = 21,658) completed pain body maps and a multi-dimensional pain assessment. Using hierarchical clustering of patients by body map selection alone, nine distinct subgroups emerged with different patterns of body region selection. Clinician review of cluster body maps recapitulated some clinically-relevant patterns of pain distribution, such as low back pain with radiation below the knee and widespread pain, as well as some unique patterns. Demographic and medical characteristics, pain intensity, pain impact, and neuropathic pain quality all varied significantly across cluster subgroups. Multivariate modeling demonstrated that cluster membership independently predicted pain intensity and neuropathic pain quality. In a subset of patients who completed 3-month follow-up questionnaires (N = 7,138), cluster membership independently predicted the likelihood of improvement in pain, physical function, and a positive overall impression of change related to multidisciplinary pain care.

**Conclusions:**

This study reports a novel method of grouping patients by pain distribution using an algorithmic approach. Pain distribution subgroup was significantly associated with differences in pain intensity, impact, and clinically relevant outcomes. In the future, algorithmic clustering by pain distribution may be an important facet in chronic pain biosignatures developed for the personalization of pain management.

## Introduction

The experience of pain is complex and personal, making it difficult to communicate and quantify. Despite these measurement challenges, parsing the experience into distinct constructs has afforded considerable progress in grouping chronic pain syndromes, defining chronic pain as a disease, and tailoring pain treatments [1, 2]. Pain quality, intensity, duration, temporal fluctuations, and regional distribution over the body are all important characteristics that are frequently queried [3]. Pain location and radiation are fundamental to chronic pain diagnosis, as outlined by multiple consensus statements [4]. The bodily distribution of pain is most commonly measured with the use of pain drawings, in which the patient or participant marks areas of their pain on a drawn figure of the body, *i*.*e*., the body map [5]. A body map is included in many validated measures of chronic pain, including the McGill Pain Questionnaire [6] and the Brief Pain Inventory [7]. Recent variations on the pain body map have improved granularity [8, 9], overlayed other pain characteristics [10], and transitioned from hand-drawn to digital maps [11–13]. In their clinical use, these tools aid experienced clinicians in quickly identifying a pattern of pain distribution that fits with a known clinical diagnosis.

Stemming from recent work on fibromyalgia, it has become clear that clinical pain syndromes thought to be distinct entities may share clinically-relevant features, especially regarding the impact of pain distribution on clinically important outcomes. A main feature of fibromyalgia is widespread pain with the most recent diagnostic criteria relying heavily on patient-reported areas of pain [14–16]. Clinical similarity to fibromyalgia, without necessarily a comorbid diagnosis, strongly influences the course and outcome of post-operative pain [17, 18] and multiple chronic pain syndromes [19–21]. In a sample of patients from primary care clinics who were not previously diagnosed with fibromyalgia, researchers have found that chronic widespread pain, defined by reaching a threshold number of painful body areas, is associated with considerably worse overall health outcomes [22]. Clinical gestalt would suggest that patterns of pain distribution would also be important, in addition to a total sum of painful body areas, since these patterns are used in current methods of diagnosis. However, to our knowledge, patterns of pain distribution have not been systematically examined as a predictor of pain characteristics, functional impact, and/or outcomes.

The goals of the current study were (1) to identify discrete patterns of patient-reported pain distribution in chronic pain patients, and (2) to determine the relationship(s) of these patterns with pain intensity, impact, and clinical outcome. If patterns are evident, they may provide additional information beyond the simple tally of painful areas. Rather than relying on prior diagnostic classifications, our approach was to utilize hierarchical clustering as a way to identify distinct groups of patients by similarities in body map selection alone. This algorithmic approach did indeed reveal several subgroups of patients in a large sample of chronic pain patients with considerable differences in pain intensity, quality, impact, and course.

## Methods

This was an observational cohort study utilizing the University of Pittsburgh’s Patient Outcomes Repository for Treatment registry (PORT) [23], which links patient-reported outcomes collected with the Collaborative Health Outcomes Information Registry software (CHOIR) [24, 25] with electronic medical record data related to appointments at University of Pittsburgh Medical Center (UPMC) Pain Medicine clinics. The University of Pittsburgh Institutional Review Board and the UPMC Quality Improvement committee approved this research with a waiver of individual informed consent.

### Patients

The study cohort consisted of 21,658 patients (3/17/2016–6/25/2019) who completed the pain body map question while completing a set of validated pain assessments as part of a clinical visit to the University of Pittsburgh’s seven pain management clinics (S1 Fig). The earliest available body map and assessment were used for each patient. A subgroup of patients (N = 7,138) completed follow-up pain assessments three months after this initial assessment. Patients presenting to UPMC Pain Medicine clinics come from a large geographic area of Western Pennsylvania and present with a variety of pain complaints. As previously described and as part of routine clinical care, patients complete validated pain assessments, including the pain body map and other validated chronic pain measures, in the waiting room prior to their clinical visit [23, 26].

### Patient demographics and other characteristics

Data from the electronic medical record (EMR) variables in PORT were used to determine age (at time of initial assessment), gender (male or female), race, payor (Medicaid or not Medicaid), comorbidity, and body mass index (BMI). Race was patient-reported and derived from the EMR as one of 17 categories which were combined due to group sizes for statistical analysis into 3 groups: White, Black, and all other races. Reporting of race is standard in the field [27] and required by funding agencies. Comorbidity was measured using the Charlson Comorbidity Index, which allows for quantification of severity of medical comorbidities [28, 29]. BMI was calculated in the EMR closest in time to the initial body map assessment with the following equation: BMI = weight (kg) / height (m)^2^.

### Patient-reported measures

Patients completed a set of validated pain assessments on a tablet using the Collaborative Health Outcomes Information Registry (CHOIR), which is an open-source, web-based software program designed for tracking of patient-reported outcomes in chronic pain patients in the context of pain management programs [24]. Pain location, pain intensity, pain interference, neuropathic quality, physical function, sleep disturbance, global physical health, anxiety, depression, and global mental health were all captured using validated instruments as described below, consistent with assessment recommendations of the Initiative on Methods, Measurement, and Pain Assessment in Clinical Trials (IMMPACT) [3, 30]. All Patient-Reported Outcomes Measurement Information System (PROMIS) instruments, except the pain intensity numeric rating scale, yield standardized T-scores normalized to a large sample of the US population (mean (M) = 50, standard deviation (SD) = 10, range Var(X) = 0–100) [31, 32]. Pain interference, physical function, sleep disturbance, anxiety, and depression were administered as Computerized Adaptive Tests, which use item-response theory to calculate T-scores thereby reducing the total number of questions required of the respondent [24, 33]. The validity of these instruments has been established through their iterative development in multiple chronic conditions [34, 35], including chronic pain (recently reviewed by Patel and colleagues [36]).

#### Pain body map

**Fig 1:**
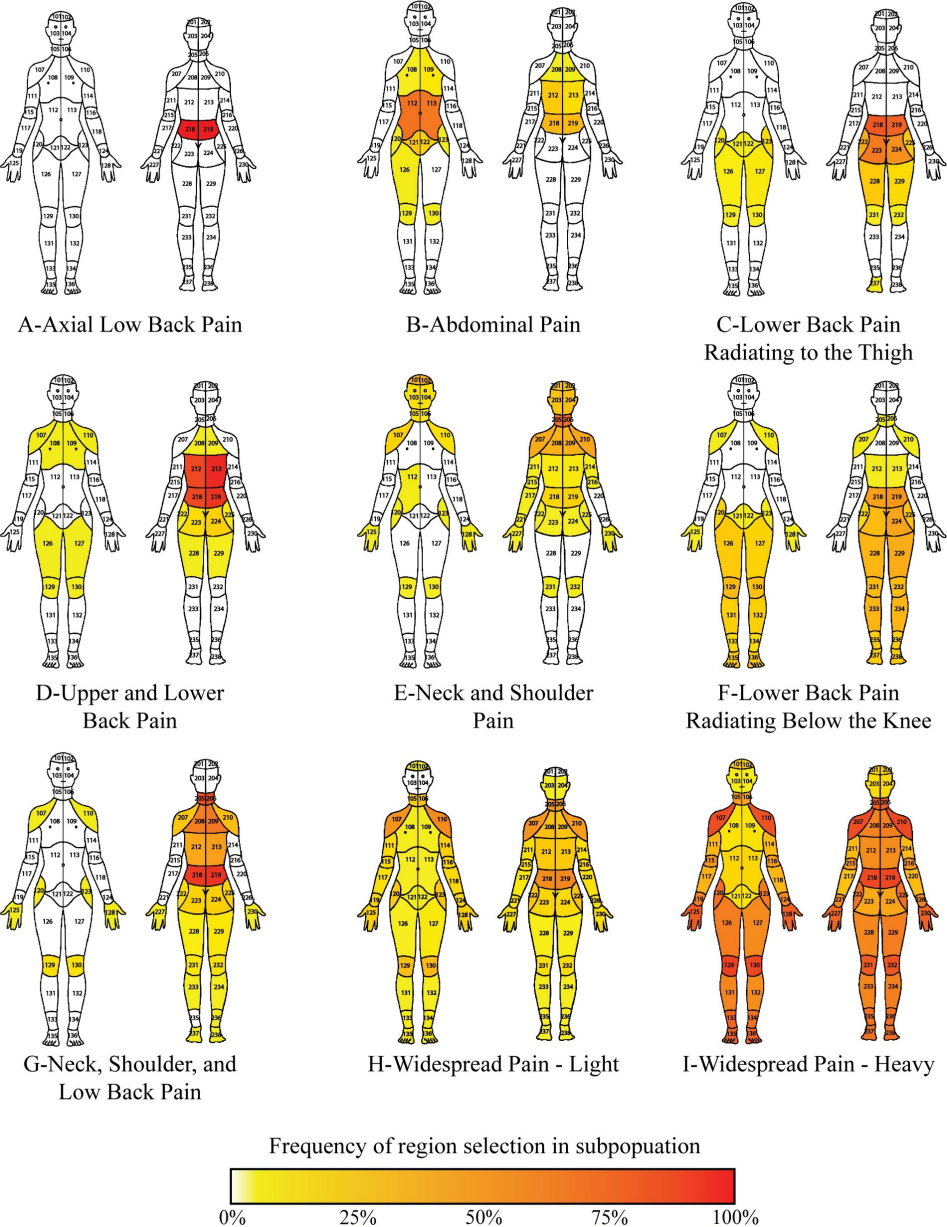
Hierarchical clustering of patient-reported body map selections reveals 9 distinct clusters. Each cluster is represented with a body map. The frequency of each region being selected within each cluster is depicted by heatmap (increasing frequency with white–yellow–red, scale at bottom).

Areas of pain are selected on two side-by-side manikins, an anterior and posterior representation of the body with lines dividing anatomically distinct body regions and a craniocaudal line dividing left and right sides as previously validated and used in multiple studies [23–26, 37–39]. The instructions were “Select the areas where you are experiencing pain” (see Fig 1). In total, there are 74 regions that may be selected. The total number of body regions reflects the simple sum of selected regions for each patient, ranging from 0 to 74. The body map presented to each patient was either female or male depending on their reported gender in the EMR. Female and male manikins were the same as previously developed and validated, showing excellent face validity, test-retest reliability, and concordance with verbal descriptors of pain location [39]. The anatomic regions were the same between female and male manikins, however six regions (arbitrary numbers 112–117) on the male body map were not labelled with the same numbers as the female map. These inconsistent labels were re-coded to match the numeric coding of the female body map. The region numbers used for hierarchical clustering of male and female patients are displayed in S2 Fig.

#### Pain intensity

Patients reported the average pain intensity in the last seven days using a standard numeric rating scale ranging from 0 (“No pain”) to 10 (“Pain as bad as you can imagine”) [23].

#### Neuropathic quality

PainDetect is a validated instrument rating the neuropathic quality of multiple pain complaints [40] that has been used in the study of several chronic pain syndromes [41, 42]. Scores range from -1 to 38.

#### Physical function

Using PROMIS bank v1.1 physical function administered via CAT [32, 33], patients reported their ability to perform day-to-day physical tasks. Individual results were converted to T-score (M = 50, SD = 10, Var(X) = 0–100). Greater values indicate greater functional capacity.

#### Depression

PROMIS bank v1.0 depression via CAT was used to measure depression [32]. Results were converted to T-score (M = 50, SD = 10, Var(X) = 0–100) with greater values corresponding to more patient-reported depression symptoms.

#### Anxiety

PROMIS bank v1.0 anxiety with CAT was used to measure anxiety [32]. Results were converted to T-score (M = 50, SD = 10, Var(X) = 0–100). A higher score reflects more patient-reported anxiety symptoms.

#### Sleep disturbance

Sleep quality was measured using PROMIS bank v1.0 sleep disturbance CAT with conversion of results to T-score (M = 50, SD = 10, Var(X) = 0–100) [32]. Greater values reflect more patient-reported sleep disturbance.

#### Pain interference

Pain-related limitations on physical, mental, and social activities, referred to as pain interference, was measured using PROMIS bank v1.1 pain interference CAT [43]. Results were converted to T-scores (M = 50, SD = 10, Var(X) = 0–100). Higher values of T-score reflect a greater pain impact.

#### Global mental and physical health

Health-related quality of life was measured in mental and physical domains using PROMIS Short Form v1.1 or v1.2 global mental and physical health measures [44]. Results were converted to T-score (M = 50, SD = 10, Var(X) = 0–100). Higher values reflect a better health-related quality of life.

#### Global impression of change

Patients reported their overall impression of change since the first clinical encounter with the pain clinic using the following Likert scale: 1 = “Very Much Worse”, 2 = “Slightly Worse”, 3 = “No Change”, 4 = “Sightly Improved”, 5 = “Very Much Improved”.

### Hierarchical clustering

Dummy variables were created for each body region and coded 0 = not selected, 1 = selected. The resulting dataset, consisting only of body map selection from the initial survey, was then used for hierarchical clustering. Hierarchical clustering is an agglomerative clustering method and can result in a tree-based representation known as a dendrogram. In the dendrogram, observations that fuse earlier will be more similar. There are two crucial steps in hierarchical clustering, *i*.*e*., determining the similarity measure and selecting the agglomeration method. Because the variables in our analysis are all binary variables (0 = body region not being selected, 1 = body region being selected), we used Jaccard index or Jaccard similarity coefficient as the similarity measure [45, 46]. The Jaccard index defined as $J(A,B)=\frac{\left|A\cap B\right|}{\left|A\cup B\right|}$, where the numerator is the intersection of two observations and the denominator is the union of two observations. The agglomeration method that we adopt is Ward’s method, which aims to minimize the total within-cluster variance. At each step in Ward’s method, the algorithm finds two clusters that will result in minimum increase in total within-cluster variance after fusing. The number of clusters was narrowed by cluster size and similarity to 9 total groups through review of dendrograms and heat maps by non-clinician investigators (A.G. and N.A.). Finally, a Pain Medicine board-certified physician (A.W.) reviewed heatmaps of body area selections for the 9-clusters to assign descriptive labels to each cluster.

### Multidisciplinary pain care

Patients presenting to the University of Pittsburgh pain management clinics undergo a comprehensive clinical assessment by fellowship-trained, board-certified Pain Medicine physicians to develop a multidisciplinary treatment plan [47]. In broad terms, the multidisciplinary treatment plan involves consideration of five treatment domains: (1) medication management, (2) pain interventions such as injections or rhizotomy, (3) rehabilitation with physical and/or occupational therapy, (4) psychological therapy, and (5) alternative and complementary approaches. In the time period studied (3/17/2016–6/25/2019), treatment plan patterns were obtained and characterized on the population level (N = 21,975). Given limitations of the current dataset, granular details of treatments and patient compliance with the prescribed treatments could not be determined.

### Statistical analysis

Data were organized, cleaned, and analyzed in Excel (Microsoft, Redmond, Washington), StataMP v14 (Statacorp, College Station, Texas), and R (“utils”, “base”, and “stats” packages; R Foundation for Statistical Computing, Vienna, Austria [48]). For continuous variables, normality was assessed graphically with histograms and q-q plots. The only variable with a particularly non-normal distribution was the total number of body regions selected, with rightward skew due to a subset of participants selecting a large total number of body regions. Total number of body regions was log-transformed by calculating the natural log of total number of body regions.

Three-month outcome variables included the continuous variables, change in pain intensity and change in body regions selected, and a dichotomized composite outcome variable. Composite outcome variables reflect clinically meaningful changes in chronic pain and pain impact [36]. A patient was classified as a treatment responder if he/she showed a 30% decrease or more in pain intensity, an increase of 3 or more in physical function T-score, and/or a response of “very much improved” on the global impression of change question [49].

Descriptive results are reported as frequencies for categorical variables and means with standard deviations or medians with interquartile ranges for continuous variables. Univariate associations were probed by calculating Pearson’s chi-squared test (categorical—categorical), t-tests (binary—continuous), 1-way ANOVA (categorical–continuous), and Pearson correlations (continuous–continuous). For the non-normal variable, total number of body regions, Spearman or Kruskal-Wallis tests were used instead. Multivariate analysis was performed with linear and logistic regressions.

## Results

### Hierarchical clustering based on pain distribution yields distinct patient subgroups

**Fig 2:**
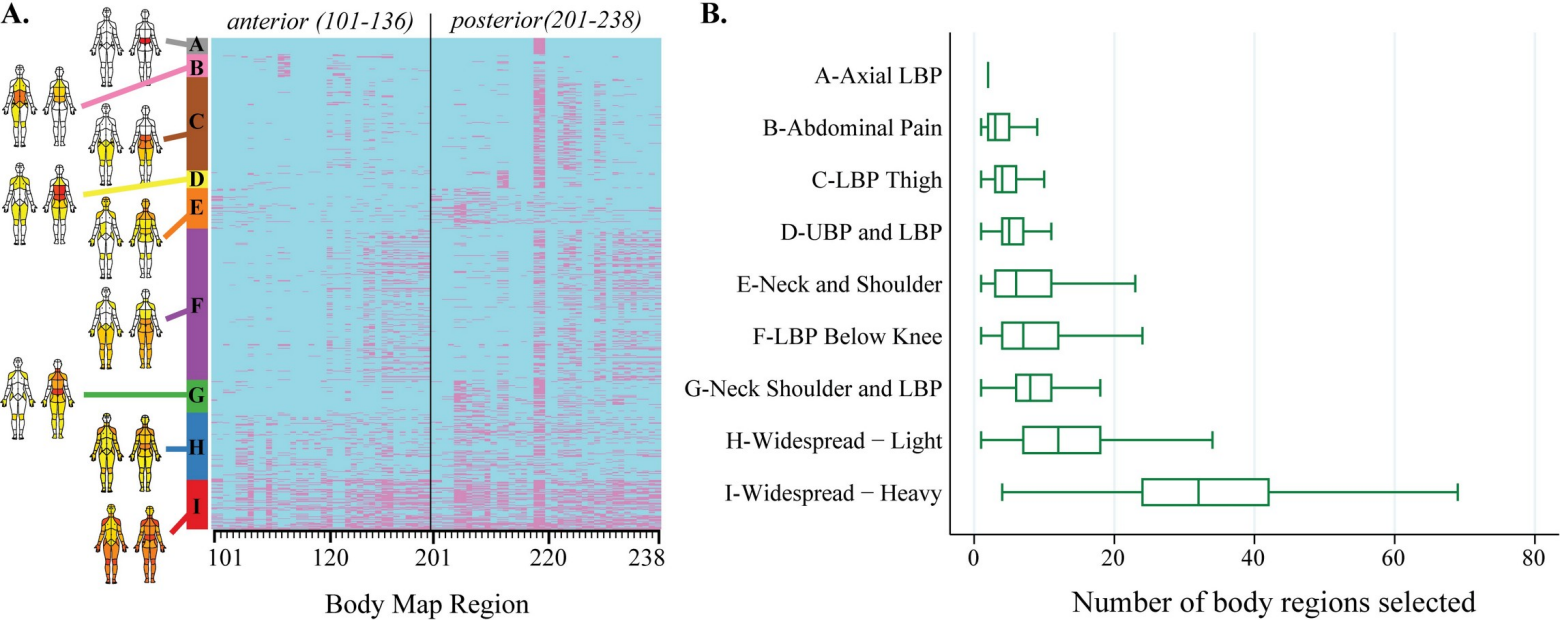
Hierarchical clustering reveals distinct subgroups despite similar total number of painful body regions. **A.** Heatmap with body map region on horizontal axis and each row on the vertical axis representing an individual patient out of the entire cohort (N = 21,658 unique patients) organized by cluster membership. **B.** Box and whisker plots representing median values (vertical line), interquartile range (boxed area), and total range (whiskers) of the simple sum of body regions by cluster membership.

Data from all patients (N = 21,658) completing a pain body map as part of a clinical visit to a large, multisite pain management practice was used in a hierarchical clustering approach, revealing 9 distinct groupings (Fig 1) according to the heatmap (Fig 2A). The frequency of body area selection within each group is depicted in Fig 1. With clinician (A.W.) review of these heatmaps, we assigned descriptive labels for each cluster based solely on the distribution of pain reported on the body map question (Fig 1).

To better appreciate the relationship between cluster assignment and a simple sum of body regions selected, patients were grouped by cluster and the selection of each body region is depicted in a heat map (Fig 2A). Although the total number of body regions differs by cluster (Kruskal-Wallis: *χ*^2^ = 8969.8, df = 8, p = 0.0001), hierarchical clustering appears to differentiate several clusters with a similar number of body regions selected. For example, inspection of box and whisker plots in Fig 2B show similar median and range values for the E-Neck and Shoulder, F-Low Back Pain (LBP) Below Knee, and G-Neck Shoulder and LBP clusters.

To estimate group differences between clusters in the total number of body regions selected, statistical modelling with one-way ANOVA was performed. In order to satisfy the normality assumption, which is essential for the ANOVA analysis, we transformed the total body regions into natural log-scaled values. Using the log-transformed total body regions, one-way ANOVA analysis was performed for a global testing of any of the clusters being different from others (F(9, 21649) = 2090.95, p < 0.0001, R2 = 0.44), which led to the post-hoc analyses for pairwise comparisons. Although every pairwise comparison was statistically significant after Bonferroni correction, the magnitude of differences was small. The median value of pairwise differences was 0.44, excluding the comparisons that involved H-Widespread–Light and I-Widespread–Heavy clusters. The pairwise comparison between G-Neck Shoulder/LBP and F-LBP Below Knee showed a difference on the natural log-scale of 0.22 (95% CI: 0.16–0.29). G-Neck Shoulder/LBP and E-Neck/Shoulder differed by 0.34 (95% CI: 0.26–0.42). The pairwise comparisons between the widespread clusters (H and I) and other clusters demonstrated larger differences. For example, the pairwise difference between I-Widespread–Heavy and C-LBP Thigh was 2.00 (95% CI: 1.94–2.06); the pairwise difference between H-Widespread–Light and C-LBP Thigh was 0.94 (95% CI: 0.88–0.99).

Next, demographic and health factors were compared across cluster assignment. Although cluster assignment through hierarchical clustering was agnostic to these variables, there were statistically significant differences between clusters in age, gender, race, Medicaid status, comorbidity, and BMI (Table 1). Groups A-Axial LBP and C-LBP Thigh were oldest, while I-Widespread—Heavy was the youngest. In the total sample, 60% of patients reported female gender, with A-Axial LBP having the most male patients (52%) and I-Widespread—Heavy having the least (28%). The proportions of each race reached statistical significance across groups, but differences were relatively small and unlikely to be clinically significant. Comorbidity measured by Charlson Comorbidity Index occurred ~10% in most groups but was less frequent in the E-Neck and Shoulder group. Cluster mean BMI for all clusters were close to the total population mean BMI of 30.6 kg/m^2^, despite achieving statistical significance.

**Table 1:** Patient characteristics by cluster membership.

	All	A	B	C	D	E	F	G	H	I	p-value^a^
Total number of patients (N^b^)	21658	677	1019	4094	786	1794	6709	1430	3000	2149	
Age, years, mean ± SD	56.6 ± 15.5	61.0 ± 16.2	51.2 ± 16.6	60.8 ± 15.3	55.6 ± 16.9	53.3 ± 15.6	58.7 ± 15.4	53.5 ± 14.2	54.2 ± 14.2	51.5 ± 13.9	<0.001
Gender, % male	40%	52%	44%	41%	46%	36%	41%	39%	38%	28%	<0.001
Race, n^b^	21517	671	1012	4070	779	1783	6667	1424	2981	2130	<0.001
White, % yes	83%	85%	83%	86%	82%	87%	83%	84%	81%	81%	
Black, % yes	15%	14%	16%	13%	16%	10%	16%	15%	18%	17%	
Other, % yes	2%	2%	2%	1%	1%	2%	1%	2%	2%	2%	
Medicaid, % yes (n^b^)	22% (21358)	14% (672)	25% (1003)	15% (4043)	26% (778)	19% (1771)	19% (6623)	28% (1405)	26% (2945)	35% (2118)	<0.001
Comorbidity (CCI≧1), % yes (n^b^)	10% (21658)	11% (677)	11% (1019)	10% (4094)	11% (786)	6% (1794)	11% (6709)	9% (1430)	9% (3000)	11% (2149)	<0.001
BMI, kg/m^2^, mean ± SD (n^b^)	30.6 ± 7.7 (16026)	31 ± 7.5 (516)	28.0± 7.2 (763)	30.9 ± 7.4 (3077)	30.6 ± 7.4 (594)	28.8 ± 6.9 (1305)	31.1 ± 7.8 (4981)	30.3 ± 7.5 (1116)	30.3 ± 7.9 (2181)	31.8 ± 8.5 (1493)	<0.001

^a^ P-values result from 1-way ANOVA (continuous) or Chi2 (categorical) tests comparing the row variable over cluster membership.

^b^ N reflects the total dataset, while n reflects the subset of N with available data. Abbreviations: A-Axial LBP, B-Abdominal Pain, C-LBP Thigh, D-Upper and Lower Back Pain, E-Neck and Shoulder, F-LBP Below Knee, G-Neck Shoulder and LBP, H-Widespread—Light, I-Widespread—Heavy, CCI (Charlson Comorbidity Index for severity of medical comorbidities), BMI (body mass index).

### Cluster membership is associated with differences in all measured domains of chronic pain

**Fig 3:**
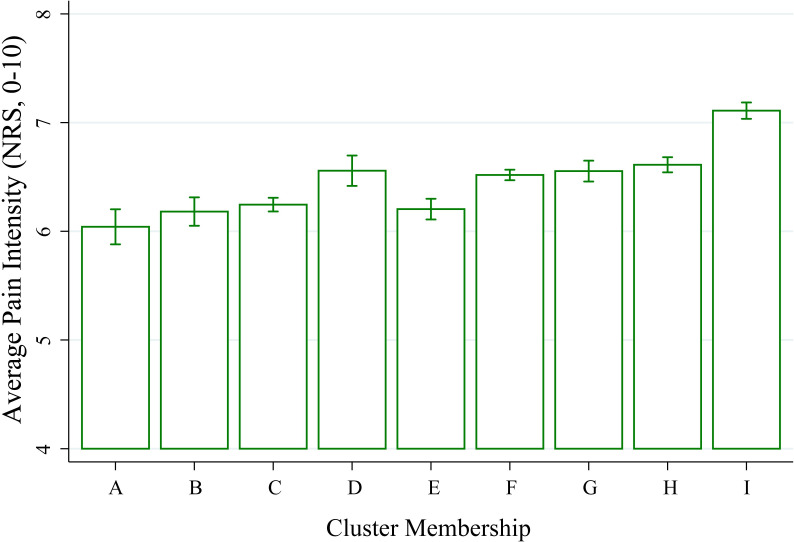
Cluster membership is associated with different average pain intensity at baseline. Histograms reflect subgroup means with error bars representing 95% confidence intervals. Total N = 21,658 unique patients. A-Axial LBP, B-Abdominal Pain, C-LBP Thigh, D-Upper and Lower Back Pain, E-Neck and Shoulder, F-LBP Below Knee, G-Neck Shoulder and LBP, H-Widespread—Light, I-Widespread—Heavy.

**Fig 4:**
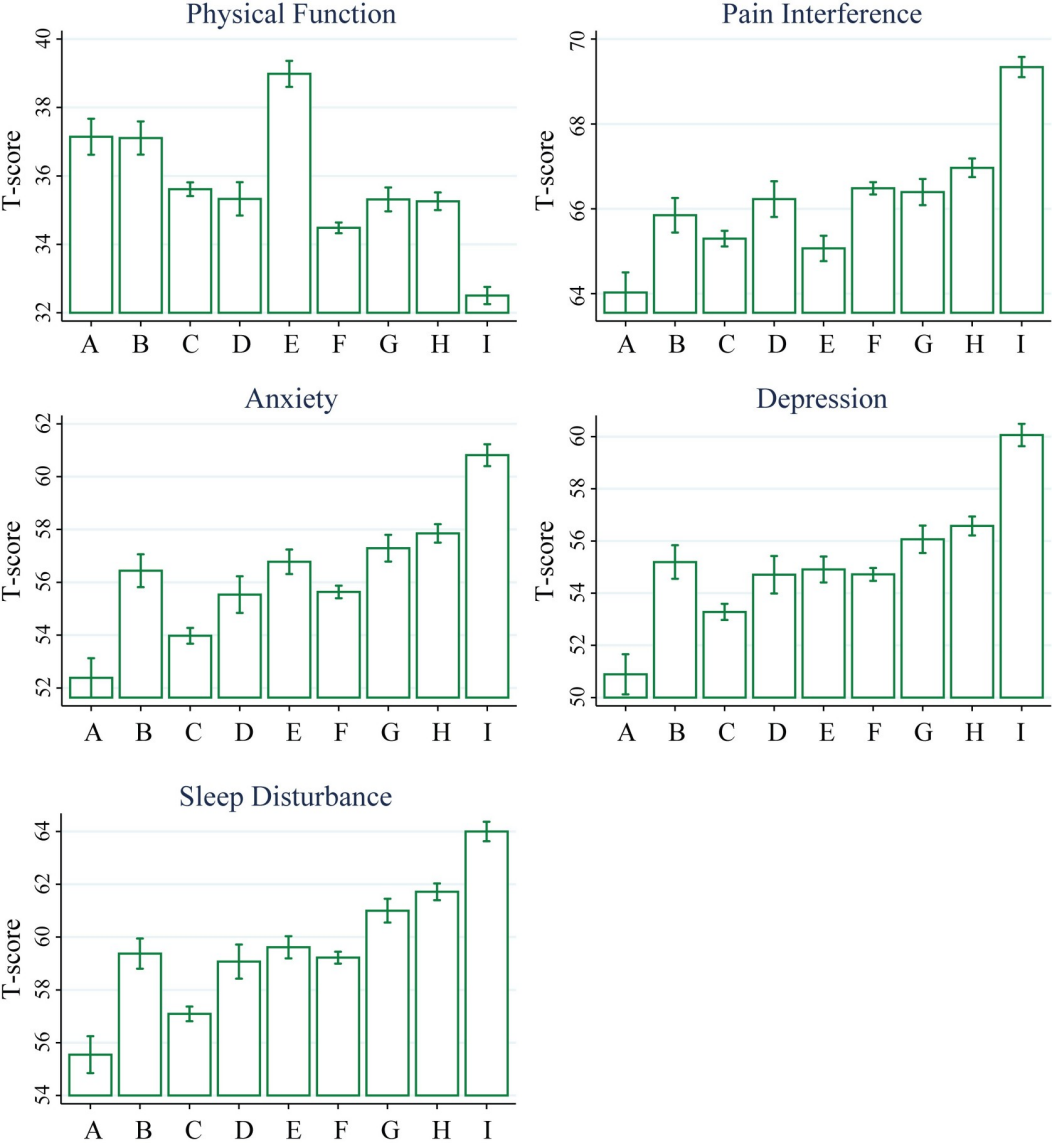
Patient-reported function, pain interference, mood, and sleep vary by cluster membership. Histograms reflect subgroup means with error bars representing 95% confidence intervals. Total N = 21,658 unique patients for physical function, N = 21,507 for pain interference, and N = 21,571 for anxiety, depression, and sleep disturbance. A-Axial LBP, B-Abdominal Pain, C-LBP Thigh, D-Upper and Lower Back Pain, E-Neck and Shoulder, F-LBP Below Knee, G-Neck Shoulder and LBP, H-Widespread—Light, I-Widespread—Heavy.

Patients who completed the body map questionnaire also completed multiple, validated instruments that reflect the multidimensional nature of pain. Data across multiple pain domains are summarized in Figs 3 and 4, and S1 Table. For each domain, statistically significant differences were detected across cluster group. Of note, cluster assignment was agnostic to these domains.

Closer inspection demonstrates that differences across cluster group occur despite similar numbers of total body region selected. Mean and 95% CI are plotted for pain intensity reported at the time of completing the body map in Fig 3. Cluster membership is associated with significantly different pain intensity (1-way ANOVA, p<0.001, S1 Table). Despite a similar number of body regions selected, the pain intensity of the E-Neck and Shoulder group is less than that of the F-LBP below knee and G-Neck Shoulder LBP groups. H-Widespread–Light appears to have similar pain intensity to other groups (e.g. G-Neck Shoulder LBP) while I-Widespread–Heavy appears to be higher than all groups.

Other domains also show differences across cluster membership (Fig 4). I-Widespread–Heavy is associated with low physical function, high pain interference, high anxiety, high depression, and high sleep disturbance. Interestingly, the second most impaired cluster is F-LBP below knee, with somewhat better physical function in the G-Neck Shoulder LBP and H-Widespread–Light groups. On the other hand, the E-Neck and Shoulder cluster is the highest functioning with relatively low pain interference. The A-axial LBP cluster is associated with relatively low anxiety, depression, sleep disturbance, and pain interference.

### Body map cluster membership independently predicts neuropathic pain quality and pain intensity

**Fig 5:**
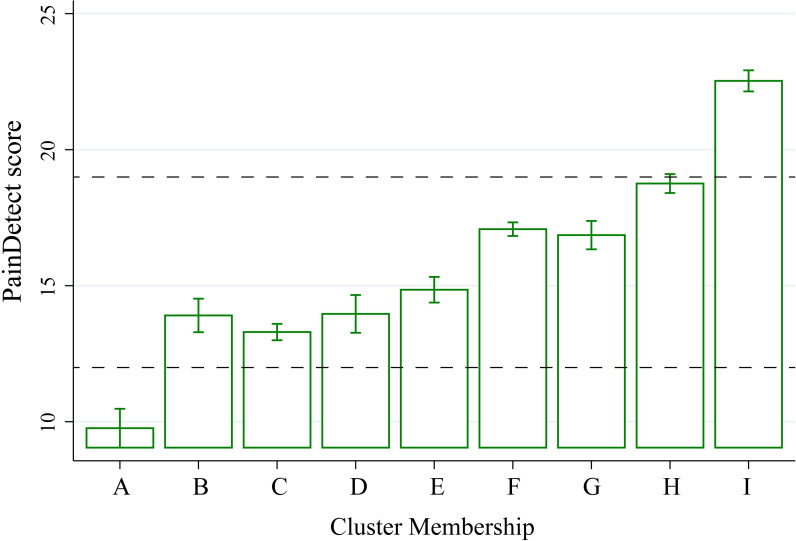
Cluster membership is associated with different neuropathic pain quality. Histograms reflect subgroup means with error bars representing 95% confidence intervals. Total N = 12,950 unique patients completed the PainDetect questionnaire, reflecting a neuropathic quality of pain. PainDetect scores 19 or greater (neuropathic),13–18 (unclear), and 12 or less (not neuropathic) are highlighted with dashed lines. A-Axial LBP, B-Abdominal Pain, C-LBP Thigh, D-Upper and Lower Back Pain, E-Neck and Shoulder, F-LBP Below Knee, G-Neck Shoulder and LBP, H-Widespread—Light, I-Widespread—Heavy.

Neuropathic pain quality measured with PainDetect also differs by cluster membership (Fig 5). Using a 1-way ANOVA to allow post-estimation testing (F(8,12941) = 231, p < 0.0001, R^2^ = 0.125), the A-Axial LBP cluster is associated with dramatically lower PainDetect scores than the F-LBP below knee cluster (-7.31, 95% CI -8.15 - -6.48), reflecting less neuropathic pain quality. The F-LBP Below Knee cluster reports considerably more neuropathic pain quality than the C-LBP Thigh cluster (3.78, 95% CI 3.39–4.17). Despite the clustering procedure being agnostic to neuropathic pain quality, there is a significant association between cluster membership and PainDetect score, with large differences noted between different low back pain groups, A-Axial LBP versus C-LBP Thigh versus F-LBP Below Knee.

Multivariate modeling was used to better understand the relationship between cluster membership and pain characteristics, including intensity and quality. First, univariate associations were calculated to identify variables to include in the model. Associations between cluster membership and outcome variables, pain intensity and PainDetect score, are noted above using 1-way ANOVA (S1 Table). In this same dataset, significant univariate associations were identified between both outcome variables (pain intensity, PainDetect) and covariates, including the sum of body regions selected, PROMIS measures, age, gender, BMI, global health measures, comorbidity, Medicaid status, and race, using Pearson’s correlations for continuous variables, t-tests for binomial variables, and 1-way ANOVA for race.

All variables with significant univariate associations were included as covariates in linear regression models, with the predictor of interest being cluster membership and the outcomes being either pain intensity (Table 2) or PainDetect (Table 3). For pain intensity, the linear regression model was statistically significant (F(22,6279) = 172.61, p < 0.0001, R^2^ = 0.38). Table 2 shows the beta coefficients for each variable in the linear regression model and p-values for each coefficient. Post-estimation with a Wald test for cluster membership was significant (F(8,6279) = 2.58, p = 0.0083), demonstrating that cluster membership significantly improved the fit of the linear regression model. Older age, female gender, Medicaid insurance, greater PainDetect, and greater scores on PROMIS measures were associated with greater pain intensity. For neuropathic pain quality, the linear regression model was significant (F(21, 6280) = 194.84, p < 0.0001, R^2^ = 0.39). Beta coefficients and corresponding p-values for each variable included in the linear regression model are listed in Table 3. Additionally, including cluster membership in the model significantly improved the fit of the model, with a statistically significant Wald test (F(8,6280) = 21.98, p < 0.0001). Younger age, Medicaid insurance, greater pain intensity, worse physical function, and more depression, anxiety, and sleep disturbance were associated with higher PainDetect scores. Of note, in both models, total number of body regions selected was included as a covariate. This suggests that cluster membership independently predicts pain intensity and neuropathic pain quality when controlling for total number of body regions selected.

**Table 2:** Linear regression model for pain intensity.

	Beta	p-value
Cluster membership (ref: A-aLBP)		
B-AbdP	-0.026	0.102
C-LBPt	-0.061	0.013
D-ULBP	0.002	0.887
E-Neck and Shoulder	-0.042	0.029
F-LBPbk	-0.077	0.008
G-NShLB	-0.019	0.289
H-Widespread–Light	-0.058	0.011
I-Widespread–Heavy	-0.04	0.098
Body regions selected	-0.043	0.007
Age	0.08	<0.001
Gender	-0.056	<0.001
BMI	-0.015	0.155
Medicaid status	0.094	<0.001
CCI	0.021	0.035
PainDetect	0.158	<0.001
PROMIS Physical Function	0.088	<0.001
PROMIS Depression	0.017	0.351
PROMIS Anxiety	0.086	<0.001
PROMIS Sleep Disturbance	0.07	<0.001
PROMIS Pain Interference	0.305	<0.001
Global Mental Health	0.311	<0.001
Global Physical Health	-0.37	<0.001
constant		<0.001

F(22,6279) = 172.61, R^2^ = 0.375

**Table 3:** Linear regression model for neuropathic pain quality.

	Beta	p-value
Cluster membership (ref: A-aLBP)		
B-AbdP	0.049	0.002
C-LBPt	0.121	<0.001
D-ULBP	0.035	0.016
E-Neck and Shoulder	0.106	<0.001
F-LBPbk	0.255	<0.001
G-NShLB	0.104	<0.001
H-Widespread–Light	0.182	<0.001
I-Widespread–Heavy	0.131	<0.001
Body regions selected	0.206	<0.001
Age	-0.077	<0.001
Gender	0.019	0.054
BMI	0.012	0.23
Medicaid status	0.066	<0.001
Pain Intensity	0.153	<0.001
PROMIS Physical Function	-0.07	<0.001
PROMIS Depression	0	0.987
PROMIS Anxiety	0.125	<0.001
PROMIS Sleep Disturbance	0.16	<0.001
PROMIS Pain Interference	0.117	<0.001
Global Mental Health	0.008	0.591
Global Physical Health	0.008	0.593
constant		<0.001

F(21, 6280) = 194.84, R^2^ = 0.393

### Body map cluster assignment on the initial assessment is associated with clinically-significant improvements in chronic pain

At the baseline assessment, Pain Medicine physicians consider multidisciplinary pain treatments for all patients, as described in Methods and operationalized in all University of Pittsburgh pain clinics [47]. The majority of patients (64.7%) seen in this timeframe (N = 21,975) were prescribed treatments from 2 or more out of 5 treatment domains: medications, interventions, physical/occupational therapy, pain psychology, complementary and integrative medicine. Patients (83.8%) were prescribed medications including anticonvulsants (47.6%), antidepressants (27.7%), nonsteroidal anti-inflammatories (48.5%), muscle relaxants (35.1%), and both strong and weak opioids (35.7% and 23.4% respectively). Interventions included lumbar (32.7%) and cervical (7.8%) spine interventions, such as epidural steroid injections, medial branch blocks, and rhizotomy. Physical and occupational therapy was prescribed in 35.8% of patients. Psychological services, including referral for pain cognitive-behavioral therapy, were prescribed in 24.5% of patients. Complementary and integrative medicine, such as acupuncture or medical cannabis, was prescribed for 3.0% of patients.

**Fig 6:**
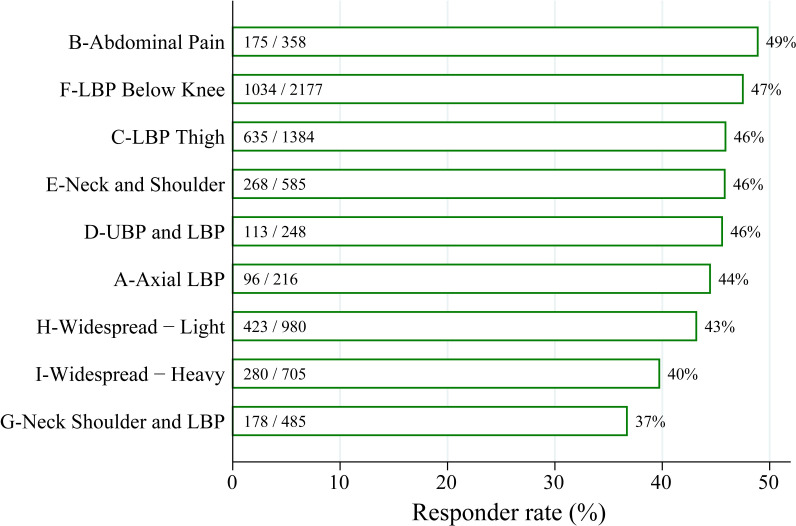
Baseline cluster membership predicts improvement in chronic pain at follow-up. Percentage of patients at 3-month follow-up who self-reported clinically significant improvements, as determined by a composite outcome consisting of improved pain, function, or a positive impression of change, are plotted by cluster membership. Proportions of patients classified as responders (n) over the total number of patients in the cluster group (N) are displayed at the base of the bars (n/N).

A subset of patients (N = 7,138) completed pain assessments 3 months after the initial assessment. Interestingly, cluster groups (established with only baseline data) reported significantly different pain intensity, physical function, pain interference, depression, anxiety, sleep disturbance, and global mental and physical health at follow-up (S2 Table). Importantly, changes in pain and changes in body area selection were also different across clusters (Table 4). Using a validated composite outcome, which included clinically significant changes in pain intensity, physical function, or impression of change, there were significant differences in response rates between cluster groups (Fig 6). Using responder rate as the outcome of interest, cluster group as the primary predictor, and baseline covariates showing a univariate association with response rate (total number of body regions, age, gender, BMI, Medicaid status, physical function, depression, anxiety, sleep disturbance, pain interference, and global physical and mental health), a multivariate logistic regression was significant (F(20,4718) = 274.41, p < 0.0001, R^2^ = 0.042). A Wald test for cluster group was significant ($\chi_{8}^{2}$ = 25.96, p = 0.0011), suggesting that body map cluster assignment at initial clinic visit independently predicts 3-month response rate. Due to limitations of the current dataset, it is not clear which multidisciplinary treatments were prescribed or the degree of patient compliance.

**Table 4:** Clinically relevant outcomes differ by cluster membership.

	ALL	A	B	C	D	E	F	G	H	I	p-value^a^
Change in pain intensity, mean ± SD (n)	-0.34 ± 1.93 (7138)	-0.22 ± 2.08 (216)	-0.48 ± 2.03 (358)	-0.36 ± 1.99 (1384)	-0.16 ± 2.02 (248)	-0.39 ± 1.78 (585)	-0.38 ± 2.05 (2177)	-0.06 ± 1.66 (485)	-0.4 ± 1.88 (980)	-0.24 ± 1.65 (705)	<0.001
Change in body regions selected, mean ± SD (n)	-0.56 ± 7.38 (7010)	1.34 ± 2.33 (209)	0.9 ± 4.49 (345)	0.61 ± 4.36 (1352)	0.4 ± 3.31 (246)	0.19 ± 6.53 (575)	-0.41 ± 6.29 (2137)	0.67 ± 6.84 (481)	-0.74 ± 8.71 (963)	-6.09 ± 12.79 (702)	<0.001

^a^ P-values result from 1-way ANOVA (continuous) or Chi2 (categorical) tests comparing the row variable over cluster membership. Abbreviations: A-Axial LBP, B-Abdominal Pain, C-LBP Thigh, D-Upper and Lower Back Pain, E-Neck and Shoulder, F-LBP Below Knee, G-Neck Shoulder and LBP, H-Widespread—Light, I-Widespread—Heavy, and Y (yes).

## Discussion

The distribution of pain in the body is a vital component of pain assessment. Apart from its utility in diagnosis, recent work has demonstrated that the extent of pain distribution affects outcomes. However, it remains unclear how patterns of pain distribution affect the pain experience. Using a hierarchical clustering approach with only a patient’s reported pain areas on a digital body map, we found multiple distinct subgroups of patients. These clusters have significantly different pain intensity, quality, and impact. Multivariate modeling suggests that cluster membership independently predicts pain intensity and neuropathic pain quality measured at the initial visit. Cluster membership based solely on body map data at the initial visit to the pain clinic significantly predicted patient-reported outcomes at 3-month follow-up, although this should be interpreted cautiously since it is unclear which specific treatments were prescribed and whether patients were compliant with recommendations. Overall, this study demonstrates that patterns of pain distribution provide information beyond a simple tally of the number of pain body regions. Given its associations with pain intensity, quality, and patient-reported outcomes, hierarchical cluster assignment based on the body map may help identify patients at risk of poor outcomes. The current study confirms the clinical relevance of pain distribution using an algorithmic approach and supports a new classification of chronic pain, termed nociplastic pain [1]. Both represent an advance towards personalized pain medicine, which shows promise in improving the treatment of chronic pain.

Subgroups of patients identified with hierarchical clustering based on body map data alone were found to be significantly different in multiple key domains, including pain intensity, pain quality, pain impact, physical function, mood, and sleep. Although the total number of body regions selected varied by cluster, cluster membership independently predicted differences in pain intensity and neuropathic pain quality. Examples of differences between clusters that might otherwise be considered a single group support this observation. Although the E-Neck and Shoulder, G-Neck Shoulder and LBP, and F-LBP below knee groups all had a similar total number of body regions selected, there were significant differences in several domains. E-Neck and Shoulder had the lowest pain intensity, highest function, and lowest pain interference among the three, and yet E-Neck and Shoulder patients reported greater anxiety than F-LBP below knee with lower depression and sleep disturbance than G-Neck Shoulder and LBP. The F-LBP below knee group had the worst physical function of the three groups but reported less anxiety, depression, and sleep disturbance than E-Neck and Shoulder and G-Neck Shoulder and LBP. This example demonstrates that cluster membership provides additional information relevant to multiple pain assessment domains.

The hierarchical clustering approach mirrors several clinically relevant scenarios. In clinical practice, distinguishing lumbar radicular pain radiating below the knee from non-radicular low back pain is important for treatment decisions, such as starting medications for neuropathic pain or considering epidural steroid injections [50]. On clinician review, three clusters seemed to reflect this common diagnostic problem for clinicians: A-Axial LBP, C-LBP thigh, and F-LBP below knee. As one might predict based on clinical assessment, these three groups varied significantly in neuropathic pain quality, measured by PainDetect. The mean PainDetect score for A-Axial LBP was 10, which signifies non-neuropathic pain [42]. For F-LBP below knee patients, the mean PainDetect was 17, which is near a cutoff of 19 for neuropathic pain. C-LBP thigh patients had an intermediate mean PainDetect score of 13, which is rated as “unclear” when differentiating neuropathic and non-neuropathic pain. This observation validates the hierarchical clustering approach by recapitulating a common clinical scenario. Of note, PainDetect scores were actually highest for the widespread pain groups (H and I). This highlights a limitation of using PainDetect, which is a screening tool. High values of PainDetect should prompt a clinical evaluation including a history probing for a neurological lesion resulting in neuroanatomically plausible symptom distribution, an examination with findings supporting the history, and confirmatory testing for a formal diagnosis of definite neuropathic pain [51]. High PainDetect scores alone should not be equated with a diagnosis of neuropathic pain.

Widespread pain was also identified by hierarchical clustering. Interestingly, two distinct groups with widespread pain emerged, I-Widespread–Heavy and H-Widespread–Light. I-Widespread–Heavy patients reported greater pain intensity, worse physical function, more pain interference, and greater anxiety, depression, and sleep disturbance than H-Widespread–Light patients. I-Widespread–Heavy also reported more painful body regions than H-Widespread–Light. Odds of a positive treatment response were also lower for the I-Widespread–Heavy compared with the H-Widespread–Light cluster. This is consistent with prior work demonstrating that a greater extent of pain is associated with worse pain and clinically meaningful treatment outcomes (e.g. [18]). Since the odds of an improvement in patient-reported outcomes at follow-up were low for both Widespread clusters compared to other clusters, this points to the utility of the body map in identifying widespread pain. Assigning cluster membership may provide additional information, allowing a quick identification of patients at highest risk (I-Widespread–Heavy) in busy clinical practice settings, such as in primary care or orthopedic practices.

An important subgroup of patients is the G-Neck Shoulder and LBP group, which has the worst odds of improvement in patient-reported outcomes at follow-up. This group has a similar total number of body regions selected as the E-Neck and Shoulder and F-LBP below knee groups but has characteristics in other domains more similar to the H-Widespread–Light group. Specifically, pain intensity, physical function, pain interference, anxiety, depression, and sleep disturbance are more similar between the G-Neck Shoulder and LBP and H-Widespread–Light groups than the E-Neck and Shoulder and F-LBP below knee groups. Since our hierarchical clustering technique identified common chronic pain syndromes, such as lumbar radiculopathy and abdominal pain, it is possible that group G represents a clinically important entity. Given similarities with H-Widespread—Light, G-Neck Shoulder and LBP may be an early stage of generalization of chronic pain that would progress into more widespread pain. Future work examining pain duration, the stability of cluster membership over time, and different pain diagnoses made for patients in G-Neck Shoulder and LBP will help test this hypothesis. If group G is an early stage of generalization, then earlier identification may lead to more appropriate treatment, such as that prescribed to patients with widespread pain. We speculate that the poor patient-reported outcomes in G-Neck Shoulder and LBP group may be due to a failure in identifying this early stage of generalization or a partially generalized phenotype, despite being evaluated by a highly trained pain physician at a university pain clinic. Group G-Neck Shoulder and LBP highlights the need for improvement in pain classification, for which body map cluster assignment may play a role in the future.

Our findings are also consistent with recent neuroscience investigations by one of our co-authors (ADW, in Ellingsen, *et*., *al*. [52]). Using functional brain MRI, this study found that a greater extent and severity of widespread pain reported on digital pain body maps (n = 79) is associated with greater disruption of resting functional connectivity between the salience network (a key brain processing network for pain) and the default mode network, even after adjusting for the effects of pain catastrophizing. The concordance in findings between these two studies is remarkable and links the neurobiology of widespread pain elucidated by functional MRI of the brain to clinical phenomenology and treatment outcomes. Thus, a case can be made that reports of widespread pain collected with digital pain body maps are *diagnostic* of pathophysiological changes in pain processing, now termed the disease of “nociplastic pain” by ICD– 11 criteria [1]. Indeed, widespread pain complaints are a central feature of nociplastic pain.

Algorithmic approaches have previously been used to identify subgroupings within identified chronic pain diagnoses. For example, hierarchical clustering analysis was used to identify quantitative sensory testing profiles that seem to cut across neuropathic pain diagnoses [53]. In fibromyalgia, clinically-important subgroupings have been identified with cluster analysis [54–56]. In a large cohort study, Backryd and colleagues used hierarchical clustering analysis in a heterogenous group of patients that included all multidimensional pain assessments [57]. The identified four subgroups did differ significantly by total body regions selected and by clinically important outcomes, although different patterns of pain distribution were not directly examined. The current study took the unique approach of applying hierarchical clustering to a very limited set of variables, *i*.*e*. the response to a single body map question at baseline. This approach allowed identification of unique patterns of pain distribution in the sample population and the important observation that these patterns impact multiple pain domains and outcomes. Moreover, identifying subgroups with the use of only one pain assessment indicates that clinically-meaningful subgroupings can be obtained quickly and easily, raising the possibility of using body map cluster membership as a screening tool to identify patients at risk of poor outcomes, even without a comprehensive psychometric assessment.

While promising, the definitive relationship of body map cluster assignment and pain pathophysiology remains to be seen. Future work will seek to identify diagnoses that fall within each body map cluster. Although this will certainly be informative, even within accepted diagnoses lies significant heterogeneity in patient characteristics. For example, in neuropathic pain, sensory profiles vary considerably within neuropathic pain diagnoses, such as lumbar radiculopathy [58]. Moreover, specific sensory profiles not commonly used to establish a diagnosis may strongly affect treatment response [59]. Recent work posits that biosignatures, combining patient-reported aspects of pain with genetics, sensory profiling, functional neuroimaging, and other measures, will allow personalization of pain diagnosis and treatment [60, 61]. Future work will explore the potential relationship of body map cluster membership assignment to sensory profiles using tools that can be feasibly incorporated into clinical practice, such as bedside quantitative sensory testing [41], to relate pain body map cluster membership to pathophysiological processes. Given its speed and ease of use for patients, we predict that body map cluster assignment will be a useful component of chronic pain biosignature development.

The current study benefits from several strengths, including a large dataset which captures patients from a large health system referred for evaluation and treatment at the only pain medicine practice in this health system. Due to the study design and nature of the dataset, causative associations cannot be established between baseline patient characteristics and follow-up outcomes. Additionally, not all patients completed pain assessments, with only a subset completing follow-up assessments. Generalizability may be limited by patient factors, including the demographics represented in the database and an outpatient Pain Medicine clinic patient sample. Outcome data do not address specific therapies, and therefore, it remains unclear which specific treatment may be helpful for a particular body map cluster. Finally, the current study focused on the use of the body map for presence of pain but did not use the body map for report of other aspects of the pain experience, such as pain quality, or accompanying symptoms, such as numbness.

## Conclusion

The current study sought to determine whether a pattern of chronic pain distribution was clinically important. Based solely on patients’ reported areas of pain on the body map, we identify distinct subgroups of patients using hierarchical clustering. Body map cluster membership determined at initial evaluation is associated with significant differences in pain intensity, pain quality, pain impact, and clinically-relevant 3-month outcomes. Certain subgroups recapitulate previously identified, clinically relevant entities (e.g. widespread pain, lumbar radiculopathy) and are related to recent findings regarding disrupted pain processing in the brain, validating the clustering approach. Prospective body map cluster assignment may be integrated in future work identifying biosignatures of chronic pain to allow for personalized pain medicine.

## Supporting information

S1 Fig Study sample flowchart. (TIF)

S2 Fig Body map region numbering used for cluster analysis. (TIF)

S1 Table Baseline pain, function, and impact differ by cluster membership. (DOCX)

S2 Table Follow-up pain, function, and impact differ by cluster membership. (DOCX)

S1 Dataset (XLSX)

S2 Dataset (CSV)

S3 Dataset (CSV)

S4 Dataset (CSV)
