# Supplementary figures and images for: Hierarchical clustering by patient-reported pain distribution alone identifies distinct chronic pain subgroups differing by pain intensity, quality, and clinical outcomes
Source: PLoS One. 2021 Aug 4;16(8):e0254862. doi: 10.1371/journal.pone.0254862 (PMC8336800; doi:10.1371/journal.pone.0254862)

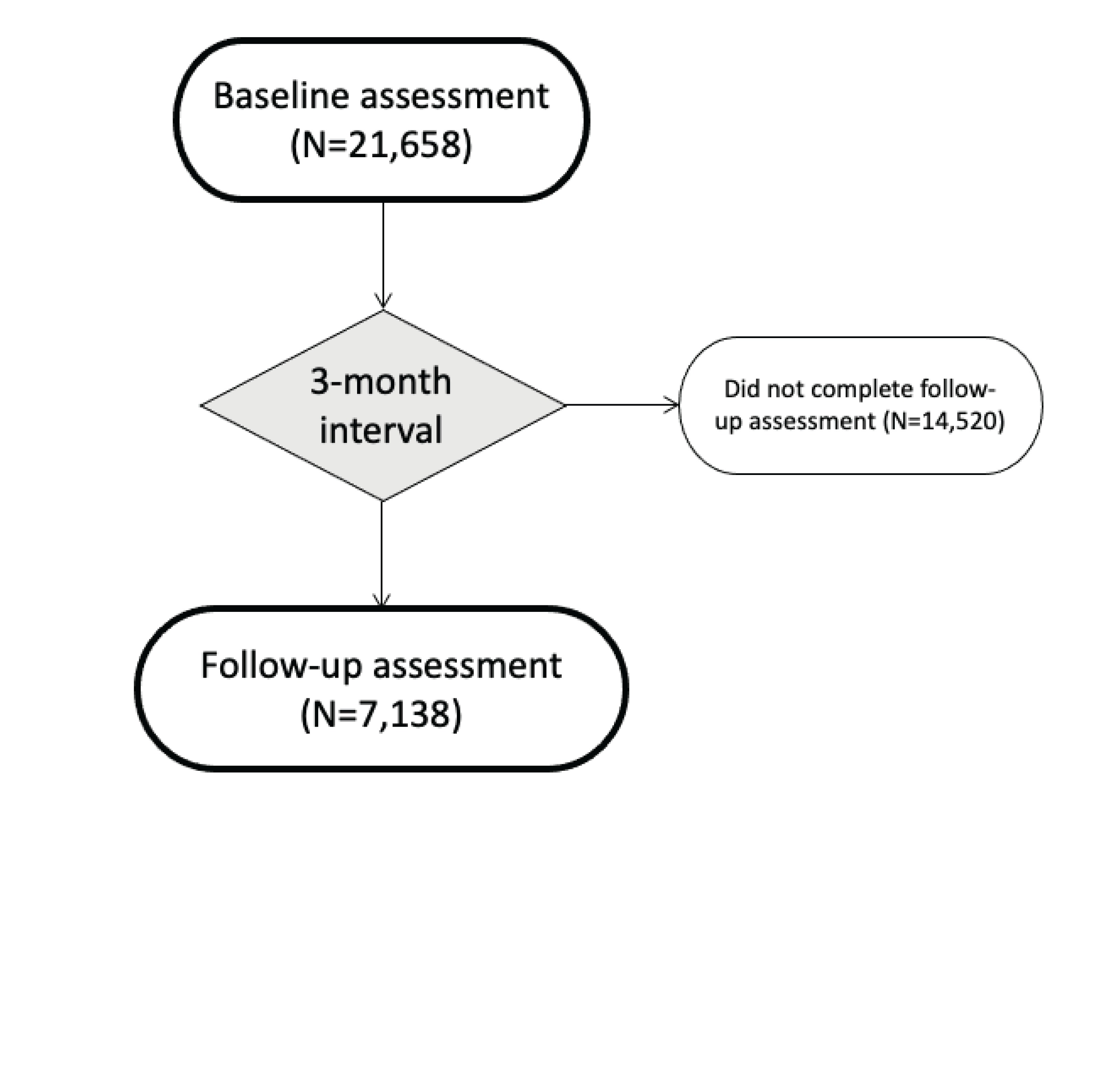

Supplement: S1 Fig — (TIF) [file pone.0254862.s001.tif]

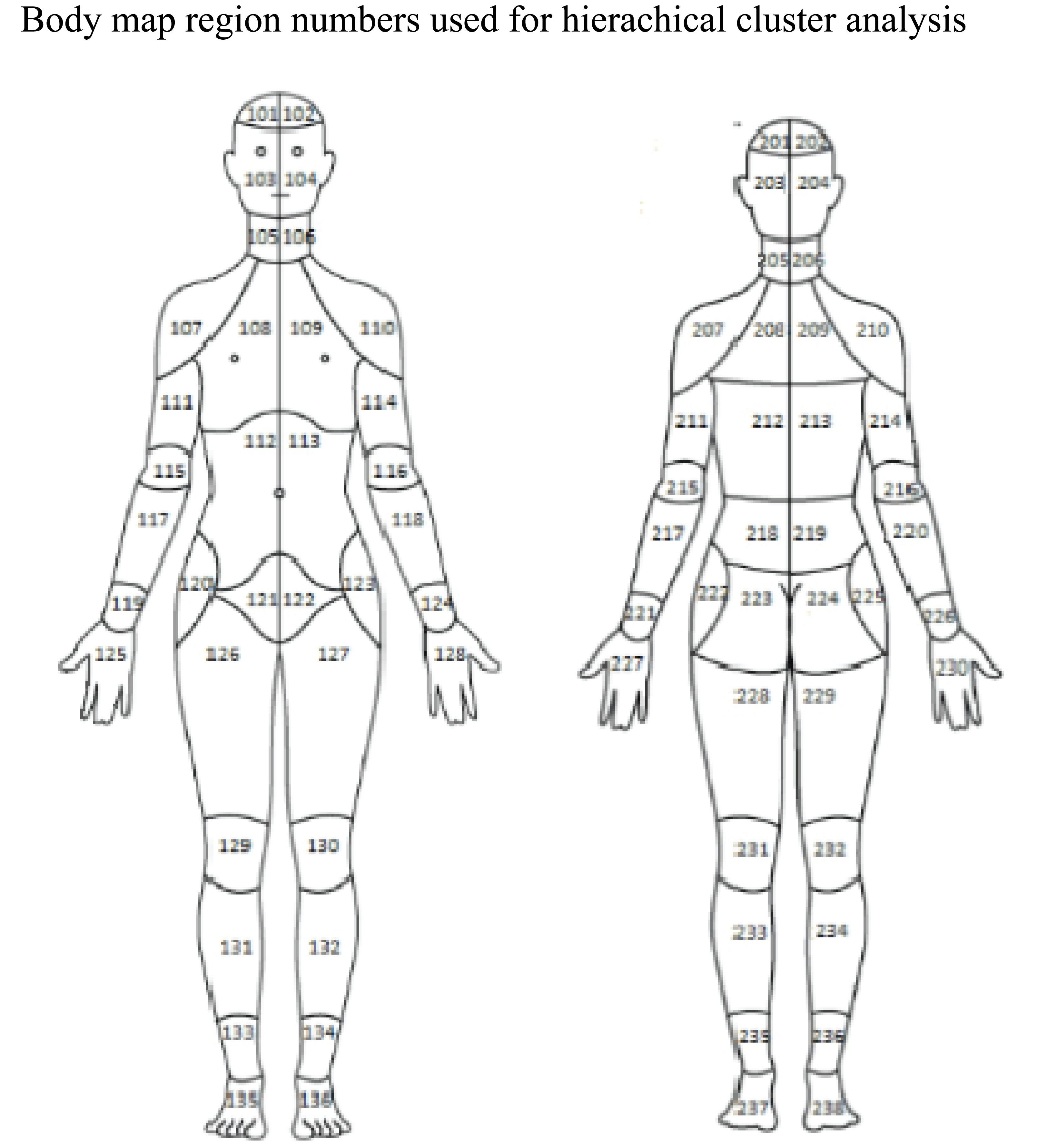

Supplement: S2 Fig — (TIF) [file pone.0254862.s002.tif]
