# Supplementary material for: Hierarchical clustering by patient-reported pain distribution alone identifies distinct chronic pain subgroups differing by pain intensity, quality, and clinical outcomes
Source: PLoS One. 2021 Aug 4;16(8):e0254862. doi: 10.1371/journal.pone.0254862 (PMC8336800; doi:10.1371/journal.pone.0254862)
Supplement: S1 Table — (DOCX) [file pone.0254862.s003.docx]

**S1 Table: Baseline pain, function, and impact differ by cluster membership.**

|  | **All** | **A** | **B** | **C** | **D** | **E** | **F** | **G** | **H** | **I** | **p-value^a^** |
| --- | --- | --- | --- | --- | --- | --- | --- | --- | --- | --- | --- |
| Total number of patients (N^b^) | 21658 | 677 | 1019 | 4094 | 786 | 1794 | 6709 | 1430 | 3000 | 2149 |  |
| Average Pain Intensity, mean ± SD | 6.49 ± 2.01 | 6.04 ± 2.14 | 6.18 ± 2.13 | 6.25 ± 2.05 | 6.56 ± 2 | 6.2 ± 2.06 | 6.52 ± 2.03 | 6.55 ± 1.85 | 6.61 ± 1.94 | 7.11 ± 1.78 | <0.001 |
| Body regions selected, mean ± SD | 10.64 ± 11.04 | 2 ± 0 | 3.94 ± 2.84 | 5.07 ± 3.01 | 5.25 ± 2.55 | 8.24 ± 7.31 | 8.64 ± 6.54 | 8.99 ± 4.08 | 13.67 ± 8.6 | 34.21 ± 14.5 | <0.001 |
| PainDetect total score, mean ± SD (n^b^) | 16.51 ± 8.26 (12950) | 9.76 ± 6.9  (361) | 13.91 ± 7.67 (597) | 13.3 ± 7.54 (2409) | 13.96 ± 7.61 (464) | 14.85 ± 7.97 (1100) | 17.08 ± 8.12 (4047) | 16.86 ± 7.58 (814) | 18.76 ± 7.57 (1819) | 22.53 ± 7.24 (1339) | <0.001 |
| *PROMIS measures:* |  |  |  |  |  |  |  |  |  |  |  |
| Physical Function, mean ± SD | 35.27 ± 7.03 | 37.15 ± 6.98 | 37.11 ± 7.91 | 35.61 ± 6.57 | 35.33 ± 6.96 | 38.98 ± 8.22 | 34.48 ± 6.6 | 35.31 ± 6.71 | 35.26 ± 7.17 | 32.51 ± 5.95 | <0.001 |
| Depression, mean ± SD (n^b^) | 55.24 ± 10.43 (21571) | 50.89 ± 10.14 (673) | 55.19 ± 10.41 (1012) | 53.28 ± 10.1 (4081) | 54.71 ± 10.23 (783) | 54.91 ± 10.69 (1788) | 54.72 ± 10.32 (6687) | 56.07 ± 10.11 (1422) | 56.58 ± 10.08 (2993) | 60.06 ± 10.1 (2132) | <0.001 |
| Anxiety, mean ± SD (n^b^) | 56.28 ± 10.05 (21571) | 52.38 ± 9.83 (673) | 56.44 ± 10.08 (1012) | 53.98 ± 9.73 (4081) | 55.54 ± 9.88 (783) | 56.78 ± 10 (1788) | 55.64 ± 9.93 (6687) | 57.29 ± 9.73 (1422) | 57.85 ± 9.72 (2993) | 60.81 ± 9.75 (2132) | <0.001 |
| Sleep Disturbance, mean ± SD (n^b^) | 59.67 ± 9.33 (21571) | 55.55 ± 9.22 (673) | 59.38 ± 9.27 (1012) | 57.09 ± 9.07 (4081) | 59.07 ± 9.22 (783) | 59.61 ± 9.05 (1788) | 59.22 ± 9.38 (6687) | 61 ± 8.67 (1422) | 61.72 ± 8.86 (2993) | 64 ± 8.71 (2132) | <0.001 |
| Pain Interference, mean ± SD (n^b^) | 66.37 ± 6.18 (21507) | 64.03 ± 6.29 (672) | 65.85 ± 6.59 (1009) | 65.3 ± 6.01 (4076) | 66.23 ± 6  (782) | 65.07 ± 6.46 (1784) | 66.48 ± 6.04 (6661) | 66.39 ± 5.93 (1417) | 66.97 ± 6.11 (2984) | 69.34 ± 5.6 (2122) | <0.001 |
| Global mental health, mean ± SD (n^b^) | 43.43 ± 9.44 (18712) | 47.27 ± 9.11 (603) | 42.89 ± 9.17 (867) | 45.3 ± 9.07 (3581) | 43.93 ± 9.2 (675) | 43.38 ± 9.55 (1546) | 44.55 ± 9.36 (5809) | 41.69 ± 8.74 (1248) | 41.96 ± 9.18 (2576) | 38.29 ± 8.94 (1807) | <0.001 |
| Global physical health, mean ± SD (n^b^) | 35.6 ± 7.18 (18712) | 38.62 ± 6.91 (603) | 36.3 ± 7.17 (867) | 36.78 ± 7 (3581) | 36.1 ± 6.88 (675) | 37.51 ± 7.68 (1546) | 35.76 ± 7.06 (5809) | 35.09 ± 6.65 (1248) | 34.7 ± 7.03 (2576) | 31.18 ± 6.08 (1807) | <0.001 |

^a^ P-values result from 1-way ANOVA tests comparing the row variable over cluster membership, except for body regions selected (Kruskal-Wallis test). ^b^ N reflects the total dataset, while n reflects a subset of N with available data. Abbreviations: A-Axial LBP, B-Abdominal Pain, C-LBP Thigh, D-Upper and Lower Back Pain, E-Neck and Shoulder, F-LBP Below Knee, G-Neck Shoulder and LBP, H-Widespread—Light, I-Widespread—Heavy
