# Supplementary material for: Hierarchical clustering by patient-reported pain distribution alone identifies distinct chronic pain subgroups differing by pain intensity, quality, and clinical outcomes
Source: PLoS One. 2021 Aug 4;16(8):e0254862. doi: 10.1371/journal.pone.0254862 (PMC8336800; doi:10.1371/journal.pone.0254862)
Supplement: S2 Table — (DOCX) [file pone.0254862.s004.docx]

**S2 Table: Follow-up pain, function, and impact differ by cluster membership**

|  | **ALL** | **A** | **B** | **C** | **D** | **E** | **F** | **G** | **H** | **I** | **p-value^a^** |
| --- | --- | --- | --- | --- | --- | --- | --- | --- | --- | --- | --- |
| Average Pain Intensity, mean ± SD (n) | 5.99 ± 2.18 (7138) | 5.52 ± 2.28 (216) | 5.47 ± 2.3  (358) | 5.69 ± 2.21 (1384) | 6.15 ± 2.18 (248) | 5.75 ± 2.22 (585) | 5.98 ± 2.21 (2177) | 6.4 ± 1.84  (485) | 6.09 ± 2.16 (980) | 6.71 ± 1.86 (705) | <0.001 |
| Body regions selected, mean ± SD (n) | 10.2 ± 10.69 (7010) | 3.34 ± 2.33 (209) | 4.8 ± 4.95  (345) | 5.98 ± 5.09 (1352) | 5.56 ± 3.85 (246) | 9.07 ± 9.31 (575) | 8.65 ± 7.77 (2137) | 9.6 ± 7.66  (481) | 13.1 ± 10.71 (963) | 26.69 ± 16.07 (702) | <0.001 |
| *PROMIS measures:* |  |  |  |  |  |  |  |  |  |  |  |
| Physical Function, mean ± SD (n) | 36.41 ± 7.2 (7138) | 37.99 ± 7.57 (216) | 39.05 ± 7.75 (358) | 36.67 ± 6.82 (1384) | 36.35 ± 7.15 (248) | 39.62 ± 8.91 (585) | 35.87 ± 6.84 (2177) | 35.41 ± 6.02 (485) | 36.42 ± 7.24 (980) | 33.8 ± 6.15 (705) | <0.001 |
| Depression, mean ± SD (n) | 53.73 ± 10.63 (6497) | 49.53 ± 9.84 (201) | 52.73 ± 10.47 (333) | 52.36 ± 10.38 (1238) | 52.35 ± 10.66 (227) | 52.95 ± 10.88 (531) | 53.14 ± 10.42 (1969) | 55.76 ± 10.6 (456) | 54.68 ± 10.55 (887) | 58.35 ± 10.21 (655) | <0.001 |
| Anxiety, mean ± SD (n) | 54.88 ± 10.32 (6497) | 51.2 ± 9.47 (201) | 54.03 ± 10.59 (333) | 53.35 ± 10.1 (1238) | 53.4 ± 10.23 (227) | 54.66 ± 10.3 (531) | 54.15 ± 10.05 (1969) | 57.33 ± 10.62 (456) | 55.9 ± 10.26 (887) | 59.12 ± 9.96 (655) | <0.001 |
| Sleep Disturbance, mean ± SD (n) | 57.64 ± 9.25 (6497) | 54.04 ± 8.97 (201) | 57.18 ± 9.3 (333) | 55.55 ± 9.08 (1238) | 56.91 ± 8.79 (227) | 57.59 ± 8.93 (531) | 57.07 ± 9.13 (1969) | 59.86 ± 9.34 (456) | 59.21 ± 9.03 (887) | 61.31 ± 8.83 (655) | <0.001 |
| Pain Interference, mean ± SD (n) | 64.52 ± 6.6 (6497) | 62.38 ± 6.79 (201) | 62.79 ± 7.57 (333) | 63.65 ± 6.28 (1238) | 63.82 ± 6.38 (227) | 63.44 ± 7.27 (531) | 64.56 ± 6.37 (1969) | 65.67 ± 5.89 (456) | 64.87 ± 6.8 (887) | 67.4 ± 5.84 (655) | <0.001 |
| Global mental health, mean ± SD (n) | 42.92 ± 9.07 (5752) | 46.86 ± 8.63 (180) | 43.02 ± 9.26 (288) | 44.32 ± 9.01 (1089) | 44.59 ± 8.02 (197) | 42.77 ± 9.01 (468) | 43.6 ± 8.83 (1759) | 41.21 ± 9.17 (407) | 41.96 ± 9.18 (788) | 39 ± 8.52  (576) | <0.001 |
| Global physical health, mean ± SD (n) | 35.87 ± 7.18 (5752) | 38.5 ± 7.46 (180) | 37.17 ± 7  (288) | 37.02 ± 7.06 (1089) | 36.98 ± 6.93 (197) | 37.32 ± 7.82 (468) | 35.93 ± 6.99 (1759) | 34.69 ± 6.51 (407) | 35.29 ± 7.27 (788) | 32.11 ± 6.16 (576) | <0.001 |

^a^ P-values were calculated by 1-way ANOVA, except for body regions selected (Kruskal-Wallis test). Abbreviations: A-Axial LBP, B-Abdominal Pain, C-LBP Thigh, D-Upper and Lower Back Pain, E-Neck and Shoulder, F-LBP Below Knee, G-Neck Shoulder and LBP, H-Widespread—Light, I-Widespread—Heavy.
